# Complete mitochondrial genome of *Compsilura concinnata* (Meigen) (Diptera, Tachinidae)

**DOI:** 10.1080/23802359.2021.1886020

**Published:** 2021-03-19

**Authors:** Yu Luo, Yan Zhi, Chuntian Zhang, Ming Yang, Jiayu Liu

**Affiliations:** a Key Laboratory of Medical Insects, Guizhou Medical University, Guiyang, China; b School of Basic Medical Science, Guizhou Medical University, Guiyang, China; c Laboratory Animal Center, Guizhou Medical University, Guiyang, China; d College of Life Sciences, Shenyang Normal University, Shenyang, China

**Keywords:** *Compsilura concinnata*, mitochondrial genome, phylogeny, Tachinidae

## Abstract

The complete mitochondrial genome of *Compsilura concinnata* (Meigen), was analyzed by next-generation sequencing approach. Its mitogenome is 15,841 bp totally, which consists of 13 protein-coding genes, 22 transfer RNA genes, two ribosomal RNA genes, and one non-coding control region. The nucleotide composition biases toward A and T, the overall A + T% was up to 80.9% of the entire mitogenome. Phylogenetic analysis supported the sister relationship between Goniini and Blondeliini. The result also suggested that the monophyly of the Exoristinae.

*Compsilura concinnata* (Meigen), belongs to the tribe Blondeliini of subfamily Exoristinae in Tachinidae where there are more than 1520 valid genera and 8500 described species around the world (O’Hara and Henderson 2020). The host range of *C. concinnata* is wide, mainly parasitizes on agricultural and forestry pests such as Phoeniidae, Danaidae, Geometridae, Hesperiidae, Lasiocampidae, Libytheidae, Lymantriidae, Noctuidae, Notodontidae, Nymphalidae, and Pyralidae (Chao et al. 2001; Shima 2006; Stireman et al. 2006).

Since the complete mitochondrial genome of *Exorista sorbillans* was reported in 2012, there are currently only seven complete mitochondrial genomes of Tachinidae have been published (Shao et al. 2012; Zhao et al. 2013; Li et al. 2017; Hou et al. 2018; Hou et al. 2019; Pei et al. 2019; Seo et al. 2019). Herein, we sequenced mitochondrial genome of *C. concinnata*, which provided sufficient molecular theoretical basis for further analysis of phylogenetic and evolutionary relationship of Tachinidae.

We describe the complete mitochondrial genome of *C. concinnata* collected from Leigong Mountain, Leishan County, Guizhou Province, China (26^○^46′24″N, 108^○^21′29″E, 2080 m) on 11 August 2020. The specimens were obtained by net catching and stored at the Key Laboratory of Medical Insects of Guizhou Medical University (accession number: CC200811). Total DNA was extracted from muscle tissues of the thorax using Rapid Animal Genomic DNA Isolation Kit (Sangon Biotech Co., Ltd., Shanghai, China). The genomic library is established and then used Illumina Hiseq PE150 platform for whole genome next-generation sequencing. The initial annotation of the mitogenome, including gene prediction and non-coding RNA, were conducted using MITOS Web Server (http://mitos2.bioinf.uni-leipzig.de/index.py) (Bernt et al. 2013; Cameron 2014). Geneious Prime 2020.2.2 was used to compare the homologous gene annotations of other insects and then submitted to NCBI (Kearse et al. 2012).

The complete mitogenome of *C. concinnata* (GenBank accession number: MW136259) 15,841 bp with double circular strands, which contains 13 protein-coding genes (PCGs), two rRNA genes, 22 tRNA genes, and one non-coding region. Four PCGs, two rRNA genes, and eight tRNA genes are distributed in the light strand among the 38 sequence elements, while others are distributed in the heavy strand. The distribution order of all elements is no different from subfamily Exoristinae in the sequence.

The 13 PCGs accounted for 70.4% of the complete mitogenome of *C. concinnata* (11,152 bp). PCGs utilize a variety of start codons including the standard ATN, except for the nonstandard TCG (*COI*). The most frequent start codon was ATG, which was covered six PCGs (*COII*, *ATP6*, *COIII*, *ND4*, *ND4L*, *CYTB*). The complete standard TAA stop codon covered 12 genes except *COII* (T) and *ND1* (TAG).

With the mitogenomic of *C. concinnata*, a phylogenetic tree was reconstructed by the maximum-likelihood method using MEGA7 with bootstrap set to 1000 (Kumar et al. 2016), the topology is shown in Figure 1. Tribe Blondeliini is sister to Goniini. High bootstrap support values of the phylogenetic tree support the monophyly of subfamily Exoristinae.

**Figure 1.**
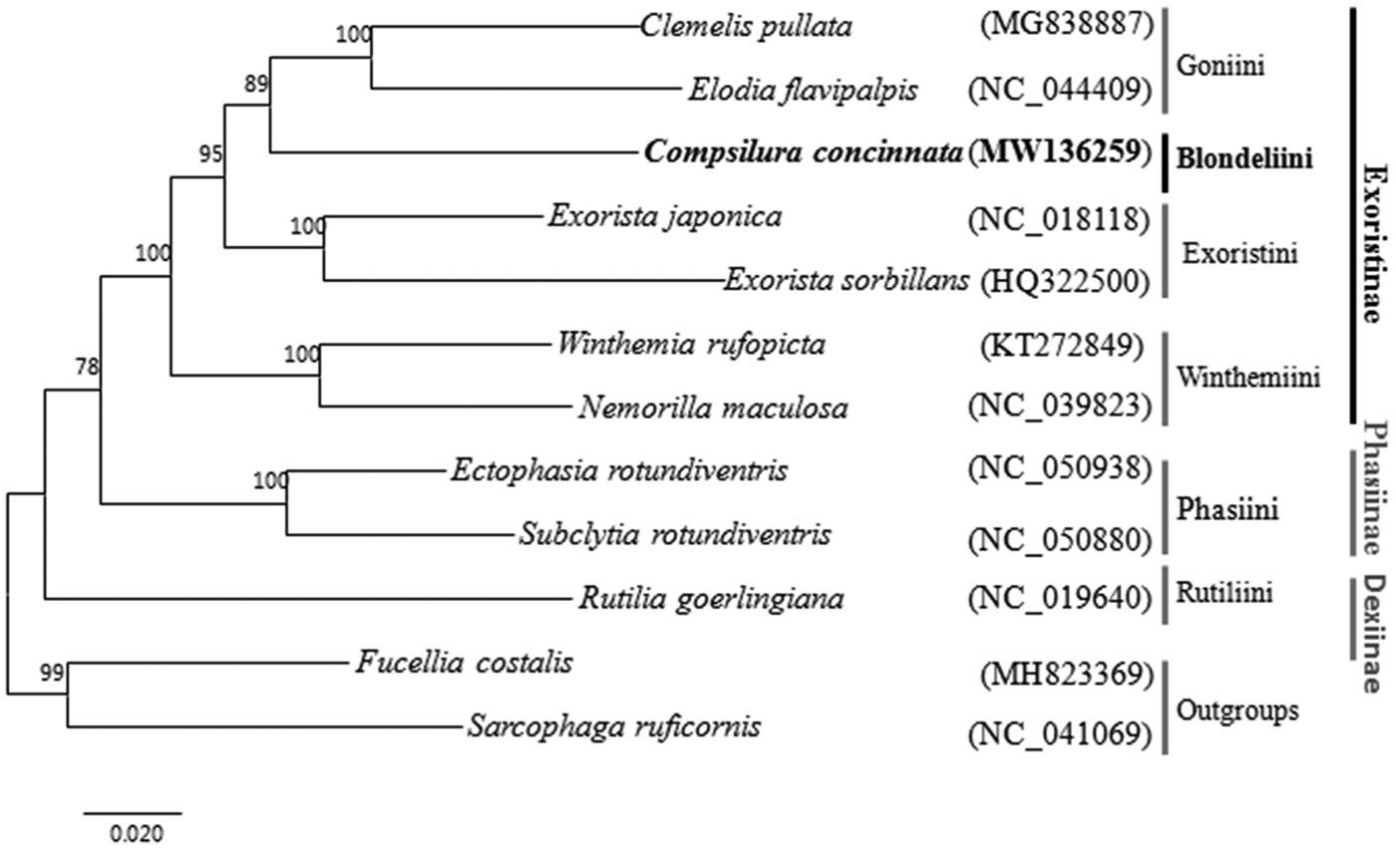
The maximum-likelihood tree was constructed based on the 13 PCGs sequence in the mitochondrial genome sequence.

## Data Availability

The genome sequence data that support the findings of this study are openly available in GenBank of NCBI at https://www.ncbi.nlm.nih.gov/ under the accession no. MW136259. The associated BioProject, SRA, and Bio-Sample numbers are PRJNA693049, SRR13480461, and SAMN17376814, respectively.
